# Supplementary material for: Green Social Prescribing in Practice: A Case Study of Walsall, UK
Source: Int J Environ Res Public Health. 2023 Sep 4;20(17):6708. doi: 10.3390/ijerph20176708 (PMC10487442; doi:10.3390/ijerph20176708)
Supplement: Supplementary file 1 [file ijerph-20-06708-s001.zip › ijerph-2463052-supplementary.pdf]

Interview questions:

Key Stakeholders (including VCSEs, Councils, funders etc.)

GSP in Walsall:

1. How many GSP programmes exist in Walsall to your knowledge?
2. How do they operate? Who funds them?
3. What resources do they have access to?
4. What communities do they serve? Or not serve?

Your organisation and role:

5. Please tell us about your organisation and its role in social prescribing in Walsall.
6. Please tell us about your role and how it is related to GSP.

Experience in delivering/supporting GSP:

7. Can you take us through your experience with GSP from the beginning to now?
8. What organisations/ people/ groups did you specifically work with as part of this role?
9. What resources, knowledge, skills etc. do you consider important in delivering your role?

Perceived impact of GSP:

10. Do you see any positive changes resulting from what you do? What are they?
11. Do you consider that GSP programmes have achieved what they were set up to achieve in Walsall?
12. Did your experience fit your expectations of GSP? Why?
13. What are the challenges?

Future of GSP:

14. How would you like to operate GSP in the future - e.g. how would you like referrals to work?
15. What change needs to happen in order for this to be achieved?
16. What impact might GSP have on your work in the community?

Users of GSP:

Introduction:

1. Tell us a bit more about yourself

Perception of GSP:

2. How long have you been with community referral? How did you come to hear about GSP?
3. What do you understand by GSP/ what does it mean to you?

Experience of GSP:

4. What organisations/ people/ groups did you specifically talk to? Any digital services or other interfaces that you interacted with?

5. Did you have more than one attempt at community referral? What were the timeframes of this?
6. What kind of support or guidance did you receive?

Impact of GSP:

7. What do you enjoy most?
8. Do you see any benefits to taking up GSP? What are they?
9. Did your experience fit your expectations of GSP? Did you face anything unexpected during your GSP experience? Is there anything you consider as barriers for you to take up GSP? (e.g.Time? Cost? Money? Travel? Resources?)

Future of GSP:

10. How would you like GSP to operate in the future to meet your needs - i.e. how would you like to be referred, or with whom would you like to interact?
11. Do you see GSP fitting in with your life? What change needs to happen in order for this to be achieved?
12. What impact might GSP have on your surrounding network, like family, friends, neighbours?
13. What does nature mean for you? What do you see it meaning for you in 20 years' time?
